# Iron regulatory protein deficiency compromises mitochondrial function in murine embryonic fibroblasts

**DOI:** 10.1038/s41598-018-23175-y

**Published:** 2018-03-23

**Authors:** Huihui Li, Hongting Zhao, Shuangying Hao, Longcheng Shang, Jing Wu, Chuanhui Song, Esther G. Meyron-Holtz, Tong Qiao, Kuanyu Li

**Affiliations:** 1 Jiangsu Key Laboratory of Molecular Medicine, Medical School of Nanjing University, Nanjing, 210093 P. R. China; 2 Laboratory for Molecular Nutrition, Faculty of Biotechnology and Food Engineering, Technion, Technion City, Haifa, 32000 Israel; 3 Department of Vascular Surgery, Drum Tower Clinical Medical College of Nanjing Medical University, Nanjing, 210008 P. R. China; 4 Present Address: Medical School of Henan Polytechnic University, Jiaozuo, 454000 P. R. China

## Abstract

Iron is essential for growth and proliferation of mammalian cells. The maintenance of cellular iron homeostasis is regulated by iron regulatory proteins (IRPs) through binding to the cognate iron-responsive elements in target mRNAs and thereby regulating the expression of target genes. *Irp1* or *Irp2*-null mutation is known to reduce the cellular iron level by decreasing transferrin receptor 1 and increasing ferritin. Here, we report that *Irp1* or *Irp2*-null mutation also causes downregulation of frataxin and IscU, two of the core components in the iron-sulfur cluster biogenesis machinery. Interestingly, while the activities of some of iron-sulfur cluster-containing enzymes including mitochondrial aconitase and cytosolic xanthine oxidase were not affected by the mutations, the activities of respiratory chain complexes were drastically diminished resulting in mitochondrial dysfunction. Overexpression of human ISCU and frataxin in *Irp1* or *Irp2*-null cells was able to rescue the defects in iron-sulfur cluster biogenesis and mitochondrial quality. Our results strongly suggest that iron regulatory proteins regulate the part of iron sulfur cluster biogenesis tailored specifically for mitochondrial electron transport chain complexes.

## Introduction

Iron is essential for growth and proliferation of mammalian cells mainly because iron is a crucial component of heme and iron-sulfur clusters (Fe-S) therefore indispensable for DNA synthesis, oxygen transport and ATP production. Iron deficiency is known to cause anaemia and permanent neurocognitive and motor impairments^1,2^. Yet, excess cellular iron could lead to the generation of reactive oxygen species that damage macromolecules such as DNA, lipid, and proteins. Excess iron in brain has been found to be associated with common neurodegenerative disorders, including Alzheimer’s, Parkinson’s, and Friedreich’s ataxia^1,3^. Therefore, organisms and cells must precisely regulate iron metabolism.

In mammals, cellular iron metabolism is regulated by iron regulatory protein (IRP) 1 and 2^4,5^. IRPs post-transcriptionally regulate the expression of target protein by binding to iron responsive elements (IREs) located within the 5′- or 3′- untranslated region (UTR) of the target gene transcripts. These transcripts mostly encode iron metabolism proteins, including the iron storage protein, ferritin, and iron import protein, transferrin receptor 1 (TfR1)^5,6^. When cells are iron-deficient, IRPs bind IREs located in the 5′-UTR of ferritin to inhibit the translation and in the 3′-UTR of TfR1 to stabilize the mRNA to facilitate iron import. When cells are iron-sufficient, IRP1 is converted to a [4Fe-4S]-containing aconitase and IRP2 is degraded through iron mediated proteasomal degradation^7–9^, which increases ferritin translation and promotes TfR1 mRNA degradation to prevent more iron absorption and avoid the excess iron-induced injury. Thus, IRPs play critical roles in cellular iron homeostasis.

The mouse models of *Irp1* and *Irp2* deficiency have been generated^10–12^. *Irp1*^−/−^ mice display polycythemia due to derepression of the Irp1-specific target mRNA of hypoxia-inducible factor 2α^13–15^. Two mouse models of global *Irp2* deficiency display dysregulation of ferritin and TfR1 and abnormal iron content in several tissues, and develop microcytic anaemia and erythropoietic protoporphyria^16,17^. Mice lacking *Irp2* also show symptoms of neurological disorders with motor neuron death^11,18,19^. The motor neuron death observed in the *Irp2*-null mice is likely caused in part by the diminished size of the ‘functional’ iron pool (compared to ‘total’) due to the reduced TfR1 expression and increased ferritin expression^20^. Importantly, the role of IRPs in mitochondrial iron homeostasis has also been uncovered^21^. Irp1 activation is thought to be critical for sustaining the mitochondrial iron supply therefore maintaining normal mitochondrial function^22^. *Irp2*-null mice showed tissue-specific iron insufficiency and compromised motor neurons and their mitochondrial function^20^. However, the exact mechanism by which IRPs sustain normal mitochondrial function has yet to be determined.

Both Fxn and IscU are essential proteins, with Fxn acting as an iron chaperone for iron delivery or an allosteric factor that modulates the cysteine desulfurase activity (see review^23^), and IscU as a scaffold protein for Fe-S cluster biogenesis. Deficiency of human FXN causes Friedreich ataxia, a neurodegenerative mitochondrial disease^24^. A muscle-specific alternative mis-splicing of human ISCU renders ISCU myopathy, also known as “myopathy with deficiency of succinate dehydrogenase and aconitase”^25^. It is widely believed that deficiency of Fe-S cluster biogenesis is causative of mitochondrial dysfunction in both above mentioned diseases and that iron subcellular mislocalization is a common phenomenon in the disorders of Fe-S biogenesis^3^. Thus, we wonder whether the biological manifestation of IRPs deficiency shares the same or part of the mechanism of Fxn or IscU deficiency.

In this study, we confirmed that murine embryonic fibroblasts (MEFs), derived from global *Irp1* or *Irp2* deficient mice, showed cellular iron starvation due to the low expression of TfR1 and the increased expression of ferritin. More importantly, we found that depletion of IRPs led to deficiencies of Fxn and IscU. Furthermore, the IRP depletion-induced deficiencies in Fxn and IscU impaired specifically Fe-S cluster-dependent mitochondrial respiratory chain activities, but not the activity of either mitochondrial aconitase or cytosolic xanthine oxidase (Xod). Our results suggest that Irp affects the Fe-S cluster biogenesis tailored specifically for mitochondrial electron transport chain (ETC) complexes.

## Results

### Irp1 or Irp2 ablation induces downregulation of the components of Fe-S cluster biogenesis machinery

**Figure 1:**
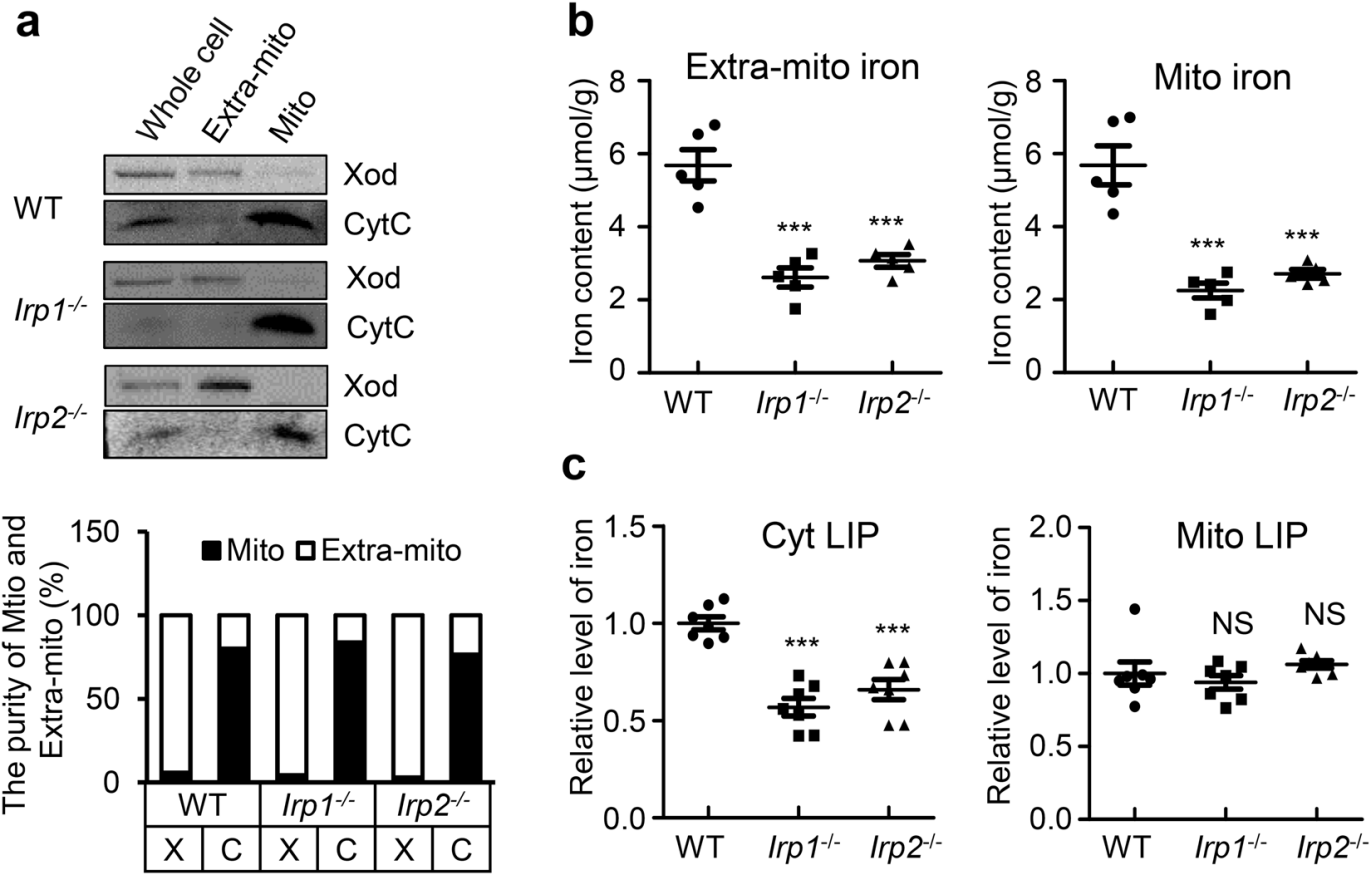
Irp1/Irp2 ablation induces cellular iron deficient, but not deficient in mitochondrial labile iron pool (LIP). (**a**) The purity of extra-mitochondrial (Extra-mito) and mitochondrial (Mito) fractions was analysed by Western blot. Xod and CytC were used as extra-mito and mito markers, respectively. A representative image set is presented. The purity of Extra-mito and Mito fractions was quantified by calculating the relative percentage of Xod or CytC in Extra-mito and Mito fractions after normalisation with total proteins (bottom panel). (**b**) Extra-mito and Mito total iron levels in mouse embryonic fibroblasts (MEFs) of *Irp1*^−/−^, *Irp2*^−/−^, and wild type (WT) measured by Ferrozine assays. (**c**) Levels of cytosolic (Cyt) and Mito LIP in MEFs of *Irp1*^−/−^, *Irp2*^−/−^, and WT measured by Calcein-AM and Rhodamine B-[(1, 10-phenanthroline-5-yl)-aminocarbonyl] benzyl ester (RPA), respectively. Values represent mean ± SEM (n = 8). A one-way ANOVA was performed. ***p < 0.001 compared with WT.

It has been reported that mice lacking Irp1 show systemic iron deficiency in the later age^13^ and lacking Irp2 show neurodegenerative symptoms^11,26^, which is thought due to the reduction of “functional iron pool”^20^. To gain further understanding of this phenotype, we used MEFs derived from global *Irp1* or *Irp2* deficient mice and measured iron content in mitochondrial and extra-mitochondrial (mito and extra-mito) fractions. First, fractionation was performed to separate mito from extra-mito fraction. Xod and cytochrome C (CytC) were used as markers to define the purity of extra-mito and mito fractions, respectively (Fig. 1a, upper panel). We quantified the purity of extra-mito and mito fractions by calculating the relative percentage of Xod or CytC in extra-mito and mito fractions after normalisation with total proteins as shown in Fig. 1a (bottom panel). Using ferrozine assays, we verified that in comparison with wild type (WT), MEFs from systemic knockout of *Irp1* or *Irp2* were iron deficient in both extra-mitochondrial and mitochondrial compartments (Fig. 1b). No significant difference between *Irp*1^−/−^ and in *Irp*2^−/−^ cells was observed. To further detect the cytosolic and mito labile iron pool (LIP, chelatable iron), we used both Calcein-AM and RPA, respectively, two well accepted iron probes, by monitoring the decay of these two fluorescent dyes. As shown in Fig. 1c, the cytosolic iron decreased significantly in both *Irp1*^−/−^ and *Irp2*^−/−^ cells comparing with WT cells, while the mito available iron content exhibited little if any changes (Fig. 1c).

**Figure 2:**
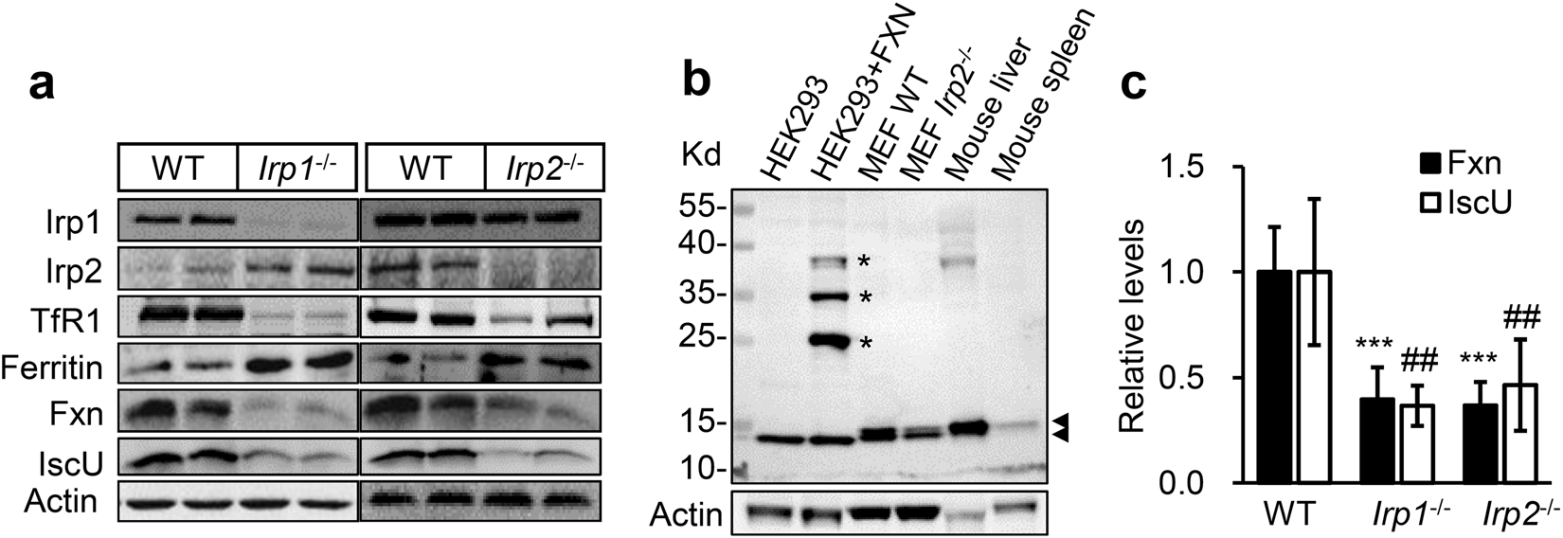
Irp1/Irp2-ablation drastically downregulates the components, Fxn and IscU, of Fe-S cluster biogenesis machinery in MEFs. (**a**) Western blot analysis of iron-related proteins including Irp1, Irp2, TfR1, Fxn, IscU, and ferritin in MEFs of *Irp1*^−/−^, *Irp2*^−/−^, and WT. Actin was used as a loading control. A representative image set is presented. (**b**) Verification of self-made FXN antibody. HEK293 + FXN: overexpressed 6×myc-tagged human full-length FXN in human cell line HEK293; Three asterisks indicate the precursor, intermediate, and mature forms of FXN; two arrows indicate the mouse and human endogenous mature forms of FXN. (**c**) Quantification of Fxn and IscU levels in MEFs of *Irp1*^−/−^, *Irp2*^−/−^, and WT. Values represent mean ± SEM (n = 3, each duplicates). A one-way ANOVA was performed. ^##^*p* < 0.01 for IscU, ***p < 0.001 for Fxn compared with WT.

Since cellular iron homeostasis is mainly controlled by the IRP/IRE machinery in mammalian cells, we verified Irps-posttranscriptionally regulated target proteins ferritin and TfR1. In comparison with WT cells, the protein level of TfR1 drastically decreased and of ferritin increased in both *Irp1*^−/−^ and *Irp2*^−/−^ cells (Fig. 2a), which is consistent with the previous studies^13,20^. These results confirm that Irp1 or 2 deficiency reduces iron uptake and increases intracellular iron chelation, which both cause cellular iron deprivation.

Iron is revealed as a key regulator of mitochondrial biogenesis^27^ and expression of Fxn and IscU is regulated by iron^28,29^, we therefore examined whether Fxn and IscU levels were altered in *Irp1*^−/−^ and *Irp2*^−/−^ cells. We first confirmed our self-made anti-Fxn antibody^30,31^, then compared Fxn and IscU protein levels among WT, *Irp1*^−/−^, and *Irp2*^−/−^ cells by immunoblotting. As shown in Fig. 2b, the antibody against Fxn was able to specifically detect the endogenous Fxn in both human and mouse samples as well as the overexpressed human FXN. When overexpressed, FXN (precursor, intermediate, and mature forms^32,33^ were clearly detected as marked with asterisks (Fig. 2b lane 2). Importantly, the levels of Fxn and IscU were markedly lower in both *Irp1*^−/−^ and *Irp2*^−/−^ cells than in WT cells (Fig. 2a), with quantification shown a more than 50% reduction (Fig. 2c). The data revealed that Irp deficiency resulted in a significant reduction in both Fxn and IscU levels.

### Downregulation of the components of Fe-S biogenesis machinery in Irp1 and 2 deficient fibroblasts is specifically associated with impaired mitochondrial respiratory chain

**Figure 3:**
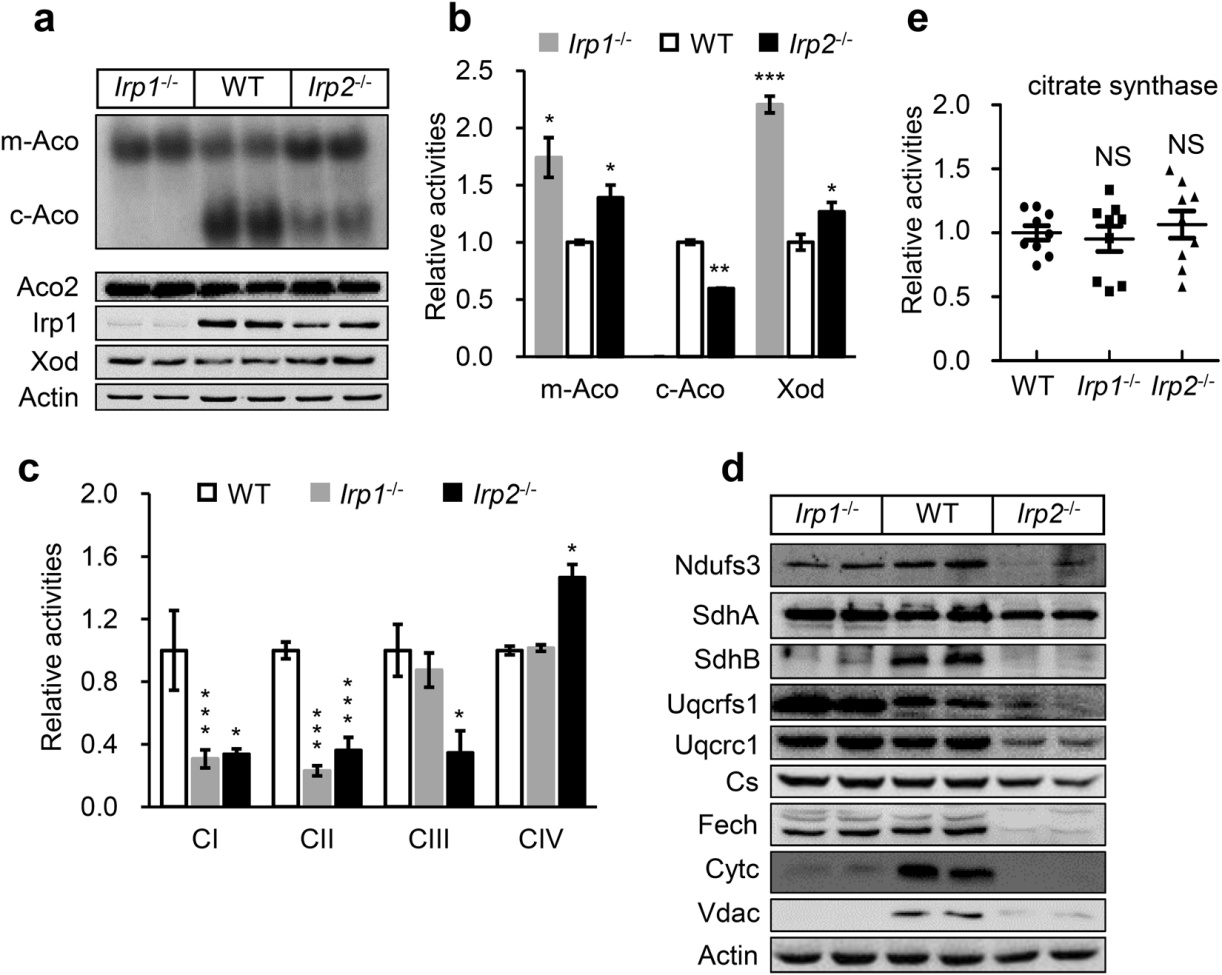
Downregulation of the components of Fe-S cluster biogenesis machinery in Irp1 and 2 deficient fibroblasts is specifically associated with impaired electron transport chain (ETC). (**a**) A representative graph of in-gel assays of mitochondrial (m-Aco, encoded by Aco2) and cytosolic (c-Aco, encoded by Irp1) aconitases in Irp deficient MEF cells (upper panel). The protein levels of Aco2, Irp1, and cytosolic Fe-S containing enzyme xanthine oxidative (Xod) were detected with western blotting. (**b**) The activities of Xod, m-Aco, and c-Aco were quantified. (**c**) Activities of ETC complexes were determined in Irp deficient MEFs. (**d**) Western blot analysis of mitochondrial proteins including Ndufs3 (a subunit of CI), SdhA and SdhB (subunits of CII), Uqcrc1 and Uqcrfs1 (subunits of CIII), Fech (matrix protein), CytC (intermembrane space protein), Vdac (outer membrane protein), and citrate synthase (Cs, matrix non-Fe-S protein). A representative image set is presented. (**e**) Activities of citrate synthase, which is a mitochondrial non-Fe-S enzyme, were determined. CI/CII/CIII/CIV: Complex I/II/III/IV. Values represent mean ± SEM (n = 10 for (**e**), n = 3, each duplicates for (**b**) and (**c**)). A one-way ANOVA was performed. *p < 0.05, **p < 0.01, ***p < 0.001 compared with WT.

Both Fxn and IscU are known as two of core components for Fe-S cluster biogenesis. Reduction of both Fxn and IscU suggests that Fe-S cluster dependent pathways were likely defective in *Irp1*^−/−^ and *Irp2*^−/−^ cells. To ascertain whether this is the case, we first measured the activities of Fe-S cluster-dependent enzyme aconitase of WT, *Irp1*^−/−^, and *Irp2*^−/−^ cells using an in-gel assay. As shown in Fig. 3a, the cytosolic aconitase activity (c-Aco) in *Irp2*^−/−^ cells was indeed decreased compared with WT (Fig. 3a), and its protein level was lower in this mutant than in WT cells. Surprisingly, no reduction was detected for mitochondrial aconitase (m-Aco) in both *Irp1*^−/−^ and *Irp2*^−/−^ cells. Instead, both activities and protein levels were clearly higher in the mutants than in WT cells. Additionally, we examined the cytosolic Fe-S cluster-containing enzyme Xod in the *Irp1*^−/−^ or *Irp2*^−/−^ cells. Similar to m-Aco described above, the protein level and activity of Xod were also higher in *Irp1*^−/−^ or *Irp2*^−/−^ cells than in WT (Fig. 3a and b). Based on these data, it appears that Irp1 deficiency has similar effects to Irp2 deficiency on some of Fe-S enzymes with increased both protein level and activity.

However, iron deficiency or downregulation of Fe-S cluster biogenesis generally brings about the low activities of Fe-S cluster dependent enzymes^25,34,35^. We wonder if the phenotype we observed above is universal for Irp deficiency. Next, Fe-S cluster-richest and dependent ETC were analysed in the *Irp1*^−/−^ and *Irp2*^−/−^ cells. We measured the activities of complex I, II, III and IV. As shown in Fig. 3c, the activities of both complex I and II in the mutant cells were only about 40% of the WT level. The activity of complex III was reduced only in *Irp2*^−/−^ cells, not in *Irp1*^−/−^ cells. Complex IV activity was at the WT level in *Irp1*^−/−^ cells and 30% higher in the *Irp2*^−/−^ cells than in WT (Fig. 3c). The negative effects of Irp deficiency are specific on ETC complexes, which are known to be functionally dependent on Fe-S clusters except complex IV.

To further analyse the effects of Irp deficiency on complex I, II, and III, we also detected the levels of complex subunits by immunoblotting. As shown in Fig. 3d, the levels of Fe-S cluster-containing subunit Ndufs3 (Complex I) and SdhB (complex II) were lower in both *Irp1*^−/−^ and *Irp2*^−/−^ cells than in the WT cells. The level of SdhA, a non-Fe-S subunit that complexes with SdhB, was higher in *Irp1*^−/−^ cells and lower in *Irp2*^−/−^ cells than in WT cells. The expression of two subunits of complex III, Uqcrc1 (not containing Fe-S cluster) and Uqcrfs1 (Fe-S cluster-containing), was markedly decreased in *Irp2*^−/−^ cells and mildly increased in *Irp1*^−/−^ cells. To see a broader effect of Irp deficiency on mitochondrial biogenesis, we detected more other mitochondrial proteins, such as ferrochelatase (Fech, a matrix protein), CytC (an intermembrane protein), and Vdac (an outer membrane protein). The protein levels of CytC and Vdac were significantly decreased in both *Irp1*^−/−^ and *Irp2*^−/−^ cells compared with WT cells (Fig. 3d). However, the level of Fech was not changed in *Irp1*^−/−^ cells and decreased in *Irp2*^−/−^ cells (Fig. 3d). For comparison, we measured the enzymatic activity and protein level for citrate synthase, a critical and non-Fe-S cluster dependent enzyme in Krebs cycle. As shown in Fig. 3d and e, the activity and protein level in both *Irp1*^−/−^ and *Irp2*^−/−^ cells were similar to those in the WT. Taken together, these results reveal that Irp deficiency, particularly *Irp2*^−/−^, mainly affects Fe-S cluster dependent protein levels and activities of respiratory chain complexes.

### The increased expression of FXN and ISCU in Irp1 and 2 deficient fibroblasts reverses the cellular iron content and restores the activities of complexes

**Figure 4:**
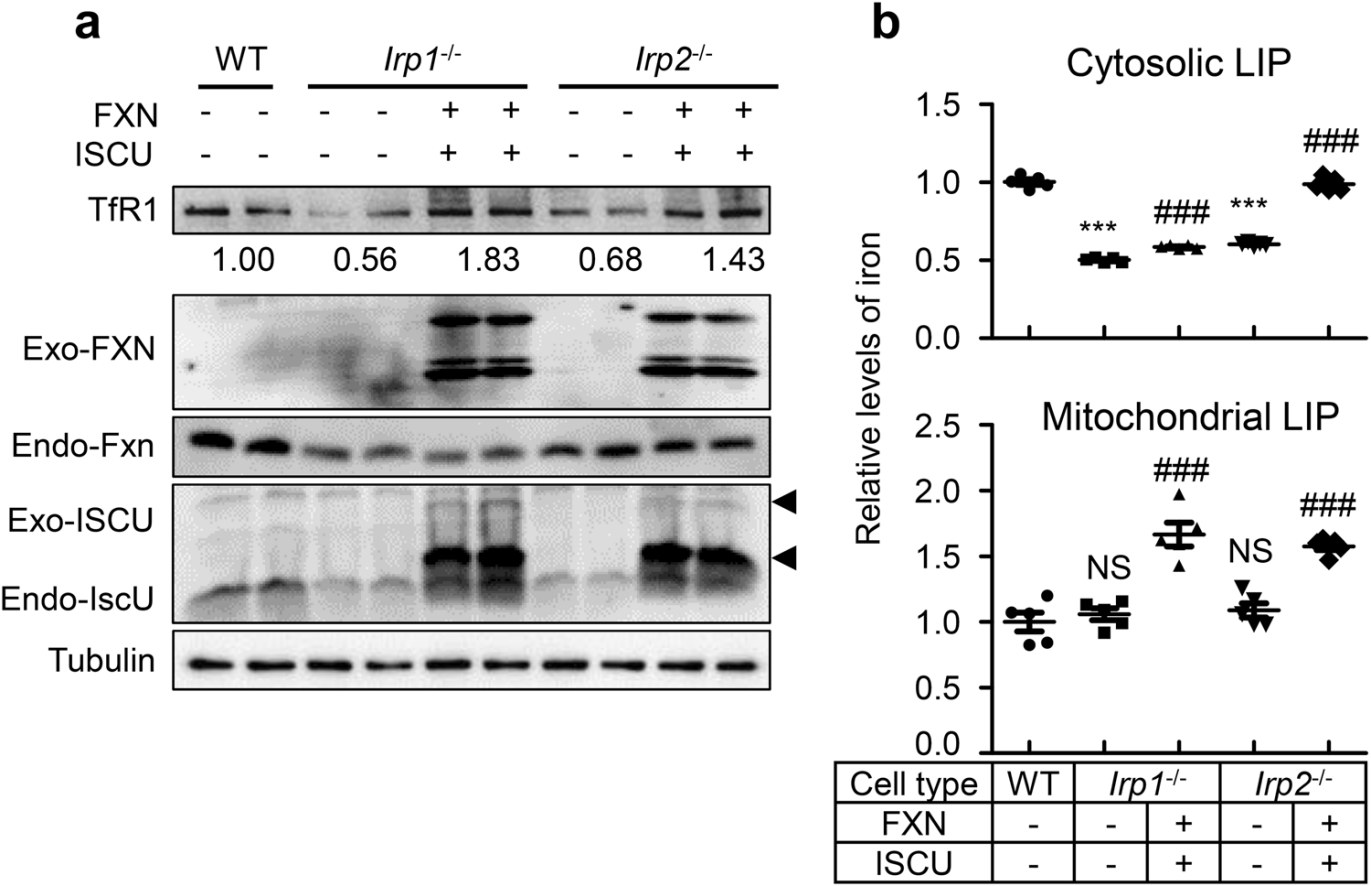
Co-expression of human FXN and ISCU in Irp deficient MEFs elevates the cellular iron content. (**a**) Western blot analysis of endogenous (Endo-) and exogenous (Exo-) FXN, ISCU, and TfR1 in MEF cells after co-transfection with pcDNA3.1-FXN-myc (encoding 6×myc-tagged human full-length of FXN) and pXS-ISCU-myc (encoding 1×myc-tagged human mitochondrial ISCU). A representative image set is presented. The arrows indicate the precursor and mature forms of exogenous ISCU. (**b**) The relative levels of cytosolic and mitochondrial LIP were measured with Calcein-AM and RPA, respectively (detail see Materials and Methods). Values represent mean ± SEM (n = 3, each duplicates for (a), n = 5 for (b)). A one-way ANOVA was performed. ***p < 0.001 compared with WT. ^###^*p < *0.001 compared the expression-plasmid transfected cells with empty plasmid transfected cells (+FXN + ISCU *vs*. -FXN-ISCU).

**Figure 5:**
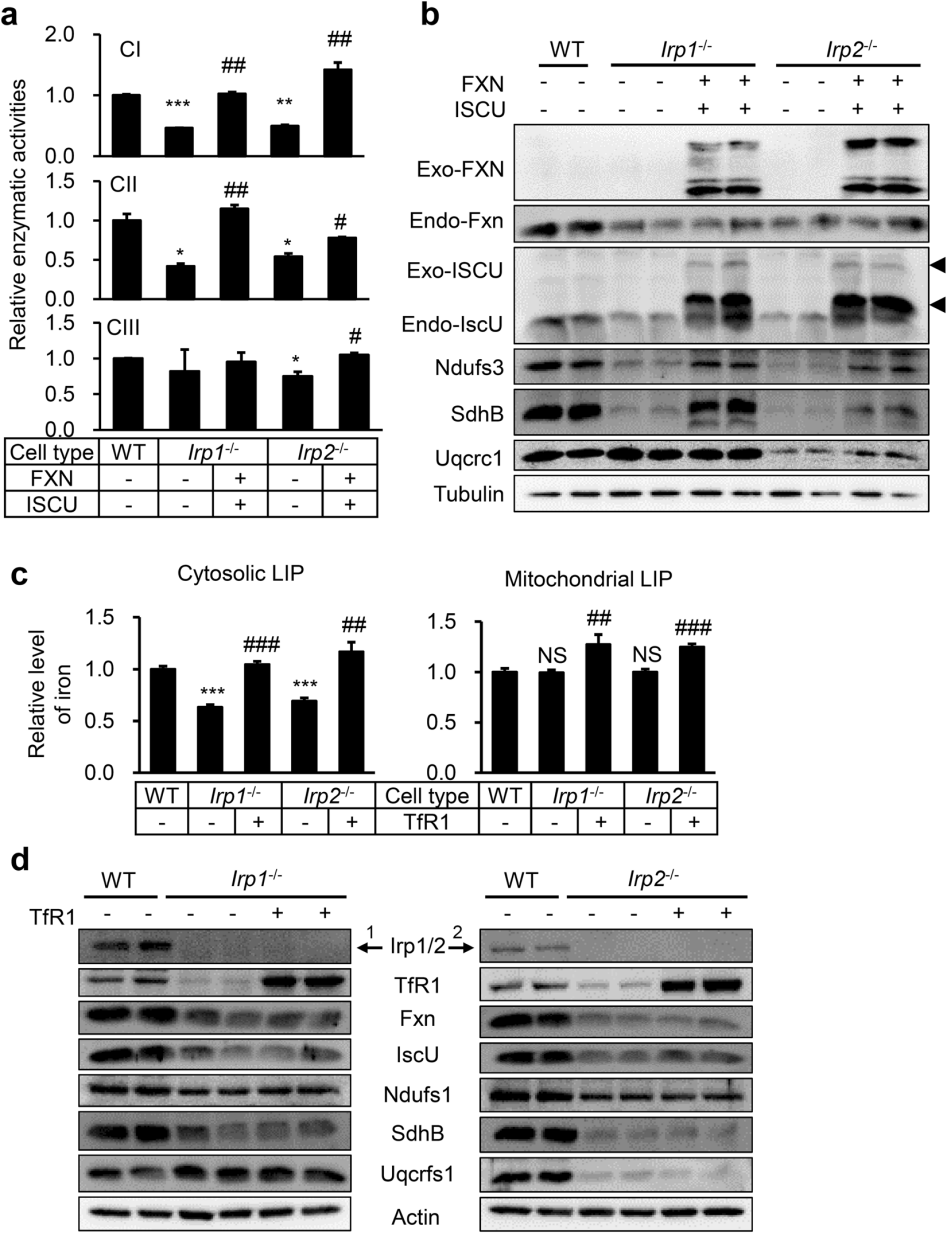
Co-expression of FXN and ISCU in Irp1 or 2 deficient MEFs rescues the mitochondrial function. (**a**) Activities of ETC CI, CII, and CIII were determined in Irp1 or Irp2 deficient MEFs after co-transfection with pcDNA3.1-FXN-myc and pXS-ISCU-myc (same plasmids as used in Fig. 4). Values represent mean ± SEM, n = 3, each duplicates. *p < 0.05, **p < 0.01, ***p < 0.001 compared with WT. ^#^*p* < 0.05, ^##^*p* < 0.01 compared + FXN + ISCU with -FXN-ISCU. (**b**) Western blot analysis of endogenous and exogenous FXN and ISCU, and mitochondrial respiratory chain proteins including Ndufs3 (a subunit of CI), SdhB (a subunit of CII), Uqcrc1 (a subunit of CIII) in MEFs after co-transfection with pcDNA3.1-FXN-myc and pXS-ISCU-myc. The arrows indicate the precursor and mature forms of exogenous ISCU. A representative dataset is presented. (**c**) The relative levels of cytosolic and mitochondrial LIP were measured with Calcein-AM and RPA, respectively. Values represent mean ± SEM (n = 3, each duplicates). ***p < 0.001 *vs*. WT. ^##^*p* < 0.01, ^###^*p < *0.001, +TfR1 *vs*. −TfR1. (**d**) Western blot analysis of iron related proteins including TfR1, Irp1 or Irp2, Fxn, IscU, and mitochondrial respiratory chain proteins including Ndufs1 (a subunit of CI), SdhB (a subunit of CII), Uqcrfs1 (a subunit of CIII) in MEFs after transfection with plasmid pcMV3-TfR1. A representative dataset is presented.

Because most of mitochondrial Fe-S cluster dependent ETC proteins are affected, we examined the role of IscU and Fxn downregulation in Irp depletion-induced malfunction of mitochondria. Human ISCU and FXN were expressed in Irp depleted cells to assess the effect on ETC basing on the conserved function of these proteins in human and rodents. Western blot analysis confirmed the successful expression of exogenous FXN and ISCU (Fig. 4a). Then, we measured the relative levels of cytosolic and mitochondrial available iron level after co-expression of FXN and ISCU. Very interestingly, both cytosolic and mitochondrial iron content increased (Fig. 4b), which was consistent with upregulated TfR1 (Fig. 4a). To determine whether overexpressed FXN and ISCU could improve the Fe-S cluster supply to mitochondrial ETC complexes in Irp deficient cells, we first measured the activities of complexes. The results showed that complex I and II activities remarkably increased in *Irp1*^−/−^ and *Irp2*^−/−^ cells (Fig. 5a, top and middle panels) and that complex III activity also increased in *Irp2*^−/−^ cells after co-expression of FXN and ISCU (Fig. 5a, bottom panel). Next, we checked whether the increased activities of ETC complexes were coordinated with the expression levels of their subunits. The levels of all tested subunits were found to significantly increase in *Irp1*^−/−^ and *Irp2*^−/−^ cells after expression of exogenous FXN and ISCU (Fig. 5b). To further proof that the decrease of Fxn and IscU was not simply due to iron deficiency, we examined whether overexpression of TfR1 would improve the mitochondrial function in *Irp1*^−/−^ and *Irp2*^−/−^ cells. Although the cytosolic and mitochondrial labile iron pool increased after overexpression of TfR1 (Fig. 5c), the protein levels of Fxn and IscU were not changed and of mitochondrial complex subunits also kept constant (Fig. 5d). These results indicate that IRPs regulate respiratory chain function likely through modulating the expression of IscU and Fxn for Fe-S biogenesis and its targeted delivery.

### The co-expression of FXN and ISCU in Irp1 or Irp2-ablation cells improves mitochondrial integrity

**Figure 6:**
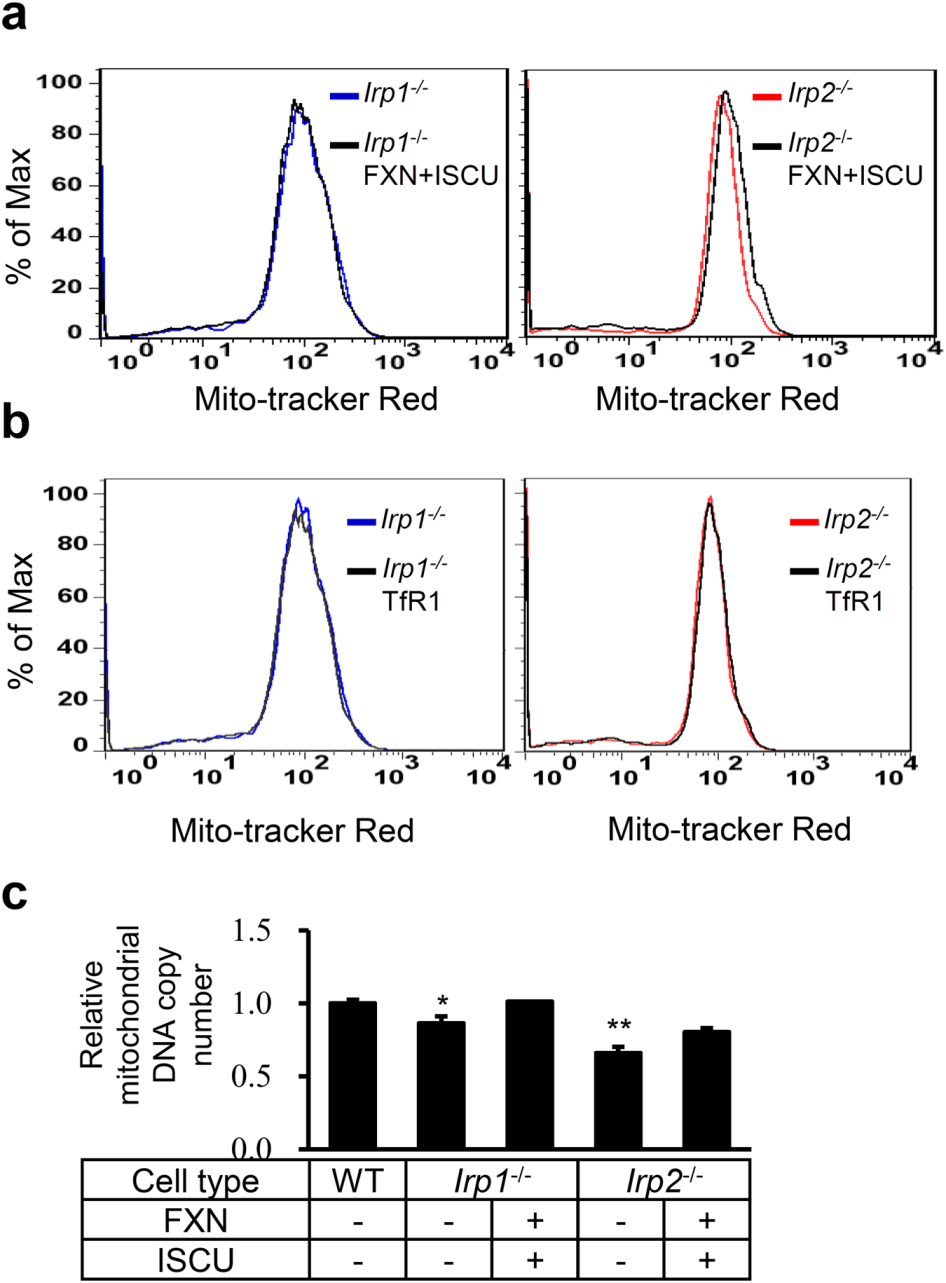
Overexpression of FXN and ISCU, but not TfR1, in Irp1 or 2 deficient MEFs improves the mitochondrial quality. MEFs were stained with Mito-tracker and analysed by Flow cytometry to represent the mitochondrial membrane potential (MMP) 24 h post transfection with plasmids pcDNA3.1-FXN-myc and pXS-ISCU-myc (**a**) or pcMV3-TfR1 (**b**). A representative graph is presented. (**c**) Relative mitochondrial DNA copy number after co-overexpression of FXN and ISCU in *Irp* ablation cells. n = 3, each duplicates. *p < 0.05, **p < 0.01 compared with WT.

To further confirm the effect of co-expression of FXN and ISCU in Irp depletion cells on mitochondria, we examined the integrity and quantity of mitochondria by measuring the intensity of mito-tracker staining and the copy number of mitochondrial DNA (mtDNA), respectively. Mito-tracker stains mitochondria in living cells and its accumulation is dependent upon mitochondrial membrane potential (MMP), a subcellular marker for mitochondrial integrity. The intensity of mito-tracker staining represents the level of MMP, revealed by Flow cytometry. The data repeatedly showed a clear right shift of the peak representing increased MMP in *Irp2*^−/−^ cells (Fig. 6a, right panel) after FXN and ISCU co-expression, but not in *Irp1*^−/−^ cells (Fig. 6a, left panel). However, this shift was not observed when TfR was overexpressed (Fig. 6b). To estimate the mitochondrial quantity, we measured the relative copy number of mtDNA presented by the ratio of mtDNA to nuclear DNA (nDNA). Irp1 or Irp2 deficiency significantly reduced the relative mitochondrial DNA copy number. We did observe the alteration of the ratio in mutant cells between before and after FXN and ISCU co-expression, but without significance (Fig. 6c). Our data indicate that enhanced mitochondrial Fe-S cluster biogenesis by increasing expression of the core components improves the mitochondrial quality and quantity in Irp depletion cells.

## Discussion

In this study, we report that *Irp1* or *Irp2*-null mutation causes the decreased expression of Fxn and IscU, two important components of Fe-S cluster biogenesis machinery. Though Irp ablation down-regulates the expression of Fxn and IscU, the enzymatic activities of Fe-S cluster-containing aconitase and Xod were not diminished. Surprisingly, mitochondrial respiratory chain is severely impaired. Moreover, increasing the expression of FXN and ISCU reversed the Irp depletion-induced deficits including cellular iron content and activities of complex I, II, and III. Our results indicate that compromised function of the respiratory chain in response to Irp-depletion could be due to the downregulation of Fxn and IscU, which specifically reduces the acquisition of Fe-S cluster by respiratory complexes in MEFs.

Irp1 and Irp2 regulate the expression of target proteins posttranscriptionally by binding to IREs in transcripts that mostly encode iron metabolism proteins for iron uptake, storage, or export^5,6^. Their targeted deletion causes changes in the levels of their direct targets including reduced levels of TfR and increased levels of ferritin in tissues^12,19,20^. In current *in vitro* study, the decreased TfR1 and increased ferritin were verified in each Irp deficient MEFs, to a severe extent in *Irp1*^−/−^ cells. Our results support the notion that both Irps are important contributors as iron regulatory proteins, giving priority to different cell types or tissues^12–15^.

Iron deprivation could strongly impair the mitochondrial respiratory chain and mitochondrial biogenesis^27,36–38^. Irp ensures adequate cellular iron supply that includes supply for proper mitochondrial function^21^, in which work both *Irp1* and *Irp2* were silenced in hepatocytes. The individual crucial roles of Irp1^22^ and Irp2^20,39^ in mitochondrial function have also been raised. Here we provide more evidence to verify the important roles of Irp1 and Irp2 for functional mitochondria. We speculate that the impaired mitochondrial function in Irp deficient cells may be caused by insufficient Fe-S cluster biogenesis due to the reduced expression of components, namely Fxn and IscU, of Fe-S synthesis machinery. Unexpectedly, the activities of mitochondrial aconitase and Xod, two Fe-S cluster-containing enzymes encoded by Aco2 and Xod, respectively, increased under normal culture condition. This could be, only partially, explained by the modestly increased protein levels of both Aco2 and Xod. Indeed, Aco2 has an IRE localized in the 5′-UTR of its mRNA^40^. Under the condition of Irp depletion-induced iron deprivation, the increased expression of Aco2 suggested that both Irps contribute to the regulation of Aco2. The increased activities of m-Aco and Xod could be also due to the increase of HIF^41,42^, one of which subunits, HIF1/2a, might be stabilized in IRP deficient cells due to the low level of iron^43,44^. However, the Fe-S cluster cofactor is essential for these enzymes to gain the enzymatic activities. Alternatively, Fe-S cluster might be stabilized under the iron deficient condition. The increased activities suggest that mitochondrial iron supply be not significantly limited in Irp depletion cells though severe cellular iron deficiency was observed. This result was confirmed by that WT and *Irp* mutants exhibited comparable available iron access in mitochondria.

To further clarify whether the decreased Fxn and IscU in Irp1 or Irp2 deficiency cells impaired other Fe-S enzymes, we detected the activities of ETC complexes, which contain several Fe-S clusters as important prosthetic groups for electron transport function. Our *in vitro* data showed significantly decreased complex I and II activities in both *Irp*-ablation cells. Similar results were previously observed *in vivo* in *Irp2*^−/−^ mice^20^. This impairment might involve HSC20, an Fe-S cluster co-chaperone protein. FXN interacts with mitochondrial HSC20, and this interaction is iron-dependent^45^. HSC20 also interacts with multiple proteins involved in Fe-S biogenesis including the ISCU/Nfs1 complex and the chaperone GRP75^45^. Recent studies have shown that HSC20 guides selection of Fe-S cluster delivery by binding to a conserved leucine- tyrosine- arginine (LYR) motif presented in specific recipient Fe-S proteins or in accessory factors that likely assist Fe-S cluster insertion into target apoproteins^46^. Majority of HSC20 interactants are the components of complex I and II, including NDUFA6 and NDUFB9, DNUFS1/7/8, NDUFV1/2, SDHAF1, SDHB and more^47,48^. These suggest that IRP-deficiency may not only impair Fe-S biogenesis but also the HSC20-mediated delivery.

In this study we also found decreased complex III activity in addition to the decreased complex I and II activities in *Irp2*^−/−^ MEFs. Different from *Irp2*^−/−^ cells, *Irp1*^−/−^ cells only showed defects of complex I and II activities. Expression of Uqcrc1 and Uqcrfs1, two subunits of complex III (non-Fe-S cluster and Fe-S cluster-containing proteins, respectively), dramatically decreased in *Irp2*^−/−^ cells, while *Irp1*^−/−^ cells even expressed slightly more Uqcrc1 and Uqcrfs1 than WT cells to maintain the complex III activity. Given the instability of proteins devoid of their Fe-S^49,50^ as for SdhB and Ndufs3 in this study due to the iron starvation, a compensatory mechanism would raise the expression of non-Fe-S cluster-containing proteins as for SdhA and Uqcrc1. This was only observed in *Irp1*^−/−^ cells, not in *Irp2*^−/−^ cells. We did not check the expression of SDHAF1, a LYR complex-II specific assembly factor, important for SDH activity by interaction with SDHB^51^. The pathogenic mutations of SDHAF1 abrogate binding to SDHB, which impairs biogenesis of holo-SDHB and results in LONP1-mediated degradation of SDHB^52^. The decreased expression of SdhA in *Irp2*^−/−^ cells might also induce downregulation of SdhB protein expression^53^. Alternatively, Irp ablation-induced iron deprivation might induce a decrease at mRNA levels of the components of complexes through dynamic alterations of histone acetylation and methylation^27,37^. Thus, *Irp* ablation not only affects cellular iron content, but also affects the expression of components of Fe-S biogenesis machinery and subunits of ETC complexes to further trigger the deficiency of complex activities. This selective effect of Irp2 depletion on mitochondrial respiratory chain might partially explain the symptoms of neurological disorders in *Irp2*^−/−^ mice^11,16,20,26^. However, overexpression of human FXN and ISCU in *Irp1*^−/−^ and *Irp2*^−/−^ cells significantly improves the mitochondrial function and recovers the deficits of ETC complex subunits. Curiously, genetic and hypoxic alterations of the microRNA-210-ISCU1/2 axis promote iron-sulfur deficiency and pulmonary hypertension^54^, the same preclinical phenotype raised from *Irp*1 depletion^13^. Irp1 activation sustains mitochondrial iron supply and function rather than driving detrimental iron overload in Fxn deficient mice^22^. These results support that the reduced expression of Fxn and IscU is involved in the effects of Irp1 and Irp2 deficiency on impaired mitochondrial function to further cause the phenotypes in *Irp1*^−/−^and *Irp2*^−/−^ mice.

Collectively, our data provide *in vitro* evidence that depletion of *Irp1* or *Irp2* downregulates the expression of Fxn and IscU and specifically compromises the activities of Fe-S cluster-containing ETC in MEFs. The current results reveal the role of Irp in securing mitochondrial function through regulating the expression of the core components, Fxn and IscU, of Fe-S cluster biogenesis machinery. Our data also imply that Irps specifically tailor Fe-S delivery for mitochondrial ETC complexes.

## Materials and Methods

### Cell culture and transfection

All media and reagents for cell culture were purchased from Invitrogen (Shanghai, China). MEFs (generously given by Dr. Tracey Rouault) derived from wild type (WT) and global *Irp1* and *Irp2* deficient mice were cultured in DMEM medium with 10% heat inactivated fetal bovine serum, 4 mM glutamine, penicillin, and streptomycin. All cells were maintained at 37 °C in a humidified atmosphere containing 5% CO_2_. For transfection, HG-Trans293^TM^ transfection reagent (Genomeditech, Shanghai, China) was used according to the supplier’s manuals. The transfected plasmids included pXS-ISCU-myc, pcDNA3.1-FXN-myc, and pcMV3-TfR1 for overexpression of ISCU, FXN, and TfR, respectively. Cells were harvested 24 h post transfection for further analysis.

### Subcellular fractionation

Mitochondria were isolated from cultured WT, *Irp1*^−/−^, and *Irp2*^−/−^ cells, respectively, with the specialized cell mitochondrial isolation kit (Beyotime, Jiangsu, China) following the manufacturer’s instructions. Briefly, cells were harvested in mitochondrial isolation buffer with PMSF and transferred to a grinder for crushing until the isolation became homogeneous. The mitochondria were isolated by differential centrifugation at 600 g and 3500 g for 10 min at 4 °C, respectively. The latter pellet was washed with PBS one time, then stored in mitochondrial lysis buffer as mitochondrial fraction. The latter supernatant was transferred into new EP tubes and centrifuged at 12000 g for 10 min at 4 °C. This supernatant was considered as extra-mitochondrial fraction.

### Ferrozine iron assays and labile iron pool (LIP) measurement

Iron content was measured using a colorimetric ferrozine-based assay with some modifications^55^. Briefly, 22 μl concentrated HCl (11.6 mol/L) was added to 100 μl cell lysate (~500 μg total protein). The mixed sample was heated at 95 °C for 20 min, then centrifuged at 12,000 g for 10 min. Supernatant was transferred very gently into fresh tubes. Ascorbate was added to reduce the Fe (III) into Fe (II). After 2 min of incubation at room temperature, ferrozine and saturate ammonium acetate (NH4Ac) were sequentially added to each tube and the absorbance was measured at 570 nm (BioTek EL x 800, Shanghai, China) within 30 min.

Labile iron was measured using the iron-sensitive probes Calcein-AM (Aladdin, Shanghai, China) and Rhodamine B-[(1, 10-phenanthroline-5-yl)-aminocarbonyl] benzyl ester (RPA, Squarix GmbH, Elbestr, Germany). Briefly, 2 × 10^5^ WT, *Irp1*^−/−^, and *Irp2*^−/−^ cells were incubated with 10 μM Calcein-AM for 10 min at 37 °C in PBS and then washed one time with PBS buffer. Cells were obtained in 100 μl PBS. Cytosolic LIP was measured using fluorescent microplate reader at 495 nm (excitation) and 530 nm (emission). For mitochondrial LIP, Cells were incubated with 10 μM RPA for 15 min at 37 °C in HBSS, then incubated with 100 μl HBSS buffer for 15 min following washing once with HBSS. Mitochondrial iron was measured at 543 nm (excitation) and 601 nm (emission) using fluorescent microplate reader.

### Western blot analysis

Proteins from lysates were prepared and resolved by 12% SDS-PAGE at 100 V, transferred for 1.5 h at 250 mA onto Nitrocellulose membranes, and analysed by immunoblotting as described previously^56^. The information for primary antibodies is as follows: anti-ferritin (cat# 69090), SdhA (cat# 137040), and SdhB (cat# 178423) from Abcam (Cambridge, MA, USA), anti-Xod (cat# 55156-1-AP), citrate synthase (cat# 16131-1-AP), aconitase 2 (Aco2) (cat# 11134-1-AP), Ndufs1 (cat# 12444-1-AP), Ndufs3 (cat# 15066-1-AP), Uqcrc1 (cat# 21705-1-AP), and Uqcrfs1 (cat# 1843-1-AP) from Proteintech Group Inc. (Chicago, IN, USA), anti-Actin (cat# BM0627) from Boster (Wuhan, China), anti-Tubulin (cat# T0198) from Sigma-Aldrich (St. Louis, MO, USA), anti-TfR1 antibody (cat# 136800) from Zymed (San Francisco, CA, USA), anti-IscU, Fech, Irp1, and Irp2, anti-Fxn (self-made)^31^, anti-CytC (cat# 1896-1) from Epitmics (Burlingame, CA, USA), anti-Vdac (cat# 4661S) from Cell Signaling Technology Inc (Shanghai, China).

### Enzymatic activities

In-gel aconitase activity assays were performed as described previously^28^. Related chemicals used were purchased from Sigma-Aldrich. The activities of complex I, II, III, and IV, Xod, and citrate synthase were measured following the manufacturer’s protocols, respectively. Purchase information is as follows: Complex I from Abcam, Complex II, Complex IV, and Citrate synthase from Comin Biotechnology Co. (Suzhou, Jiangsu, China), Complex III from Biovision Inc. (Milpitas, CA, USA), Xod from Nanjing Jiancheng Bioengineering Institute (Nanjing, China).

### Flow cytometric analysis

WT, *Irp1*^−/−^, and *Irp2*^−/−^ cells were incubated with Mito-tracker Red for 5 min at room temperature in PBS and then washed twice with PBS. Cells were harvested, pelleted by centrifugation and resuspended in PBS. The fluorescence intensity was measured by flow cytometry (BD, Franklin, NJ) and data were analysed using Flowjo software.

### Quantitative real-time PCR (qRT-PCR)

Total DNA was prepared with QiaAmp DNA mini kit (Qiagen, Germantown, MD) according to the manufacturer’s instructions. qRT-PCR experiments were performed with SYBR Green PCR master mixture (Thermo Fisher Scientific). The relative copy number of mtDNA was determined by comparing the copy number of mtDNA-locating *CytB* to that of nDNA-locating *Act*. The used primer sequences (5′–3′) were: TTCATGTCGGACGAGGCTTA and CTGTGGCACCTCAGAATGAT for mouse *CytB*; ATAAGTGGCCTTGGAGTGTG and GTACGACCAGAGGCATACAG for mouse *Act*.

### Statistical analysis

The values were expressed as mean ± SEM from, at least, three independent experiments. A one-way analysis of variance (ANOVA) was carried out using SPSS ver. 22.0 software (IBM Corporation, Armonk, NY, USA). Significance was considered at *p* < 0.05.

### Data availability

The datasets generated during and/or analysed during the current study are available from the corresponding author on reasonable request.
